# Engaging Mood Brain Circuits with Psilocybin (EMBRACE): a study protocol for a randomized, placebo-controlled and delayed-start, neuroimaging trial in depression

**DOI:** 10.1186/s13063-024-08268-6

**Published:** 2024-07-03

**Authors:** Joshua M. Poulin, Gregory E. Bigford, Krista L. Lanctôt, Peter Giacobbe, Ayal Schaffer, Mark Sinyor, Jennifer S. Rabin, Mario Masellis, Amit Singnurkar, Christopher B. Pople, Nir Lipsman, Muhammad I. Husain, Joshua D. Rosenblat, Xingshan Cao, Bradley J. MacIntosh, Sean M. Nestor

**Affiliations:** 1 Hurvitz Brain Sciences Program, Sunnybrook Health Sciences Centre, Toronto, ON Canada; 2 Dr. Sandra Black Centre for Brain Resilience and Recovery, Toronto, ON Canada; 3 Department of Medical Biophysics, University of Toronto, Toronto, ON Canada; 4 Department of Neurological Surgery, University of Miami Miller School of Medicine, Miami, FL USA; 5 Harquail Centre for Neuromodulation, Hurvitz Brain Sciences Program, Sunnybrook Health Sciences Centre, Toronto, ON Canada; 6 Department of Psychiatry, University of Toronto, Toronto, ON Canada; 7 Department of Pharmacology and Toxicology, University of Toronto, Toronto, ON Canada; 8 Division of Neurology, Department of Medicine, Sunnybrook Health Sciences Centre, University of Toronto, Toronto, ON Canada; 9 Rehabilitation Sciences Institute, University of Toronto, Toronto, ON Canada; 10 Division of Neurosurgery, Department of Surgery, Sunnybrook Health Sciences Centre, University of Toronto, Toronto, ON Canada; 11 Department of Medical Imaging, Sunnybrook Health Sciences Centre, University of Toronto, Toronto, ON Canada; 12 Campbell Family Mental Health Research Institute, Centre for Addiction and Mental Health, Toronto, ON Canada; 13 Mood Disorders Psychopharmacology Unit, Poul Hansen Family Centre for Depression, Toronto Western Hospital, University Health Network, Toronto, ON Canada; 14 Research Design and Biostatistics, Sunnybrook Research Institute, Toronto, ON Canada

**Keywords:** Psilocybin, Psychedelics, Major depressive disorder, Depression, Functional magnetic resonance imaging, Arterial spin labelling, Cerebral blood flow, Randomized controlled trial

## Abstract

**Background:**

Major depressive disorder (MDD) is a leading cause of disability worldwide across domains of health and cognition, affecting overall quality of life. Approximately one third of individuals with depression do not fully respond to treatments (e.g., conventional antidepressants, psychotherapy) and alternative strategies are needed. Recent early phase trials suggest psilocybin may be a safe and efficacious intervention with rapid-acting antidepressant properties. Psilocybin is thought to exert therapeutic benefits by altering brain network connectivity and inducing neuroplastic changes that endure for weeks post-treatment. Although early clinical results are encouraging, psilocybin’s acute neurobiological effects on neuroplasticity have not been fully investigated. We aim to examine for the first time how psilocybin acutely (intraday) and subacutely (weeks) alters functional brain networks implicated in depression.

**Methods:**

Fifty participants diagnosed with MDD or persistent depressive disorder (PDD) will be recruited from a tertiary mood disorders clinic and undergo 1:1 randomization into either an experimental or control arm. Participants will be given either 25 mg psilocybin or 25 mg microcrystalline cellulose (MCC) placebo for the first treatment. Three weeks later, those in the control arm will transition to receiving 25 mg psilocybin. We will investigate whether treatments are associated with changes in arterial spin labelling and blood oxygenation level-dependent contrast neuroimaging assessments at acute and subacute timepoints. Primary outcomes include testing whether psilocybin demonstrates acute changes in (1) cerebral blood flow and (2) functional brain activity in networks associated with mood regulation and depression when compared to placebo, along with changes in MADRS score over time compared to placebo. Secondary outcomes include changes across complementary clinical psychiatric, cognitive, and functional scales from baseline to final follow-up. Serum peripheral neurotrophic and inflammatory biomarkers will be collected at baseline and follow-up to examine relationships with clinical response, and neuroimaging measures.

**Discussion:**

This study will investigate the acute and additive subacute neuroplastic effects of psilocybin on brain networks affected by depression using advanced serial neuroimaging methods. Results will improve our understanding of psilocybin’s antidepressant mechanisms versus placebo response and whether biological measures of brain function can provide early predictors of treatment response.

**Trial registration:**

ClinicalTrials.gov Identifier: NCT06072898. Registered on 6 October 2023.

**Supplementary Information:**

The online version contains supplementary material available at 10.1186/s13063-024-08268-6.

## Administrative information

Note: the numbers in curly brackets in this protocol refer to SPIRIT checklist item numbers. The order of the items has been modified to group similar items (see http://www.equator-network.org/reporting-guidelines/spirit-2013-statement-defining-standard-protocol-items-for-clinical-trials/).

**Table:** 

Title {1}	Engaging Mood Brain Circuits with Psilocybin (EMBRACE): a study protocol for a randomized, single-center, placebo-controlled, delayed-start, neuroimaging trial in depression
Trial registration {2a and 2b}.	EMBRACE Trial. ClinicalTrials.gov Identifier: NCT06072898. Registered on 6 October 2023.
Protocol version {3}	Version 5 Dated June 2, 2024
Funding {4}	This trial has research seed funding from the Sunnybrook Foundation, with further support from the Canadian Institutes of Health Research (CIHR) Project Grant (165981).
Author details {5a}	^1^Hurvitz Brain Sciences Program, Sunnybrook Health Sciences Centre, Toronto, Ontario, Canada ^2^Dr. Sandra Black Centre for Brain Resilience and Recovery, Toronto, Ontario, Canada ^3^Department of Medical Biophysics, University of Toronto, Toronto, Canada ^4^University of Miami, Florida, United States ^5^Harquail Centre for Neuromodulation, Hurvitz Brain Sciences Program, Sunnybrook Health Sciences Centre, Toronto, Ontario, Canada ^6^Department of Psychiatry, University of Toronto, Toronto, Ontario, Canada ^7^Department of Pharmacology and Toxicology, University of Toronto, Ontario, Canada ^8^Division of Neurology, Department of Medicine, Sunnybrook Health Sciences Centre, University of Toronto, Toronto, Ontario, Canada ^9^Rehabilitation Sciences Institute, University of Toronto, Toronto, Ontario, Canada ^10^Division of Neurosurgery, Department of Surgery, Sunnybrook Health Sciences Centre, University of Toronto, Toronto, Ontario, Canada ^11^Department of Medical Imaging, Sunnybrook Health Sciences Centre, University of Toronto, Toronto, Ontario, Canada ^12^Campbell Family Mental Health Research Institute, Centre for Addiction and Mental Health, Toronto, Ontario, Canada ^13^Mood Disorders Psychopharmacology Unit, Poul Hansen Family Centre for Depression, Toronto Western Hospital, University Health Network, Toronto, Ontario, Canada ^14^Research Design and Biostatistics, Sunnybrook Health Sciences Centre, Toronto, Ontario, Canada
Name and contact information for the trial sponsor {5b}	Sunnybrook Research Institute, Hurvitz Brain Sciences Program, Sunnybrook Health Sciences Centre, University of Toronto, Toronto, Ontario, Canada.
Role of sponsor {5c}	The sponsor did not have any role in the collection, management, analysis, and/or interpretation of the data; writing the report, or the decision to submit the report for publication.

## Introduction

### Background and rationale {6a}

Major depressive disorder (MDD) is a leading cause of global disability [1] with individuals facing a relative risk of all-cause mortality 1.7 times greater than the general population [2]. This heterogenous disorder is characterized by episodes of low mood and/or anhedonia, varying in severity and duration, accompanied by a spectrum of symptoms that include psychomotor changes, concentration difficulty, neurovegetative symptoms, suicidal ideation, and impaired functioning [3, 4]. MDD pathogenic effects on large-scale brain networks and alterations in functional connectivity [5–12] is an advancing area of study. Typical treatments include pharmacotherapy, psychotherapy, and therapeutic brain stimulation [13]. Conventional antidepressants (i.e., SSRIs, SNRIs) normalize functional connectivity in key emotion regulation regions [5] and act on glutamatergic and serotonergic neurotransmission [14] to improve executive function [15]. However, those with MDD who are treated with conventional antidepressants experience low remission rates (around 20–42%), a higher incidence of side effects, daily administration, and a delayed therapeutic response that can span weeks [16–20]. This underscores the need to identify alternative antidepressant therapies and to explore new potential mechanisms of antidepressant action.

Psychedelics, particularly psilocybin, are emerging as a promising antidepressant alternative and, at the time of writing, there were > 30 registered clinical studies investigating the safety and efficacy of psilocybin to treat MDD alone. Phase II clinical trials demonstrate that psilocybin is an effective antidepressant comparable to, or even potentially superior to, conventional antidepressants with encouraging results in clinical trials [16, 21–23]. Previous studies have emphasized the lasting antidepressant effects of acute psilocybin treatment with psychotherapy [4, 24], demonstrating both rapid (within days) and enduring (up to 3 months) effects [25, 26], accompanied by alterations in select brain networks for days to weeks [27]. It also exhibits a well-established safety profile with low toxicity and dependence potential [28–30]. Specifically, psilocybin has been shown to induce acute changes in cerebral blood flow (CBF) when measured acutely with arterial spin labelling (ASL) after psilocybin exposure in healthy adults [31, 32], particularly in regions associated with the default-mode network (DMN) [33]. Modulation in the DMN, a network of interconnected brain regions active during resting states and self-referential processes [34], is implicated in MDD, characterized by a global elevation in DMN resting state functional connectivity [35]. In particular, CBF reductions have been found in DMN hubs, such as the posterior cingulate cortex (PCC) and medial prefrontal cortex (mPFC) [33], occurring within hours of psilocybin macro-dosing. As such, it is pertinent to examine DMN regions to characterize regional, group, and session effects associated with psilocybin treatment.

The neurobiological mechanisms underpinning psilocybin’s antidepressant response remain unclear but likely involve changes in neuroplasticity at both cellular [36, 37] and connectomic level [37–39]. In this context, *neuroplasticity* refers to the brain’s ability to adapt structurally and functionally in response to psilocybin [40–42]. Previous research in MDD suggests increased dynamic functional activity and neural flexibility measured by BOLD-fMRI and magnetic resonance spectroscopy [43] during the subacute treatment phase (days to weeks post-dosing). However, the acute effects (e.g., < 24 h post-dosing) of psilocybin-evoked neuroplasticity in MDD remain unknown. Moreover, psilocybin’s agonism at serotonin 5-hydroxytryptamine (5-HT) receptors and its role as a glutamatergic agent are implicated in its neuroplastic mechanisms [44–47] and psychological phenomena such as ego dissolution (i.e., the subjective experience of a breakdown in one’s sense of self and separation between oneself and the world) [43, 48, 49], dream state-like cognitive processing [12, 49], and putative molecular antidepressant mechanisms [36, 50–52].

Placebo effects are well-documented in conventional antidepressant trials in the absence of acute treatment effects [53, 54]. Spontaneous remission of untreated MDD symptoms can occur for many reasons but may take longer compared to antidepressant treatments [55–57]. A recent phase 2 trial comparing psilocybin to an active placebo alongside supportive psychotherapy found significant benefits of psilocybin over placebo on Montgomery-Åsberg Depression Rating Scale (MADRS) and Sheehan Disability Score measures up to 6 weeks post-treatment [58]. However, no difference in MADRS was found at 2 days post-treatment, suggesting psilocybin’s neurobiological effects occur before symptom improvement. This underscores the need to investigate acute brain network changes during psilocybin treatment versus placebo to better understand how psilocybin immediately alters functional connectivity and the neurophysiological mechanisms linked to mood changes in MDD. This exploration may aid in identifying early treatment response markers [54]. For this study, the “acute” timeline will denote any changes that manifest on the day of psilocybin treatment, whereas the “subacute” timeline will encompass any change that occur during the subsequent weeks of follow-up.

Functional neuroimaging is ideal for assessing the acute and subacute effects of psilocybin therapy due to its ability to track changes in brain regions and circuits over treatment. Non-invasive imaging approaches like BOLD contrast and ASL provide reliable whole-brain measures without the need for intravenous contrast agents [59, 60]. BOLD and ASL image contrast share similar information to gold-standard invasive positron emission tomography approaches [61, 62], limiting the need for more costly exogeneous radiotracers. ASL-CBF imaging, offering quantitative maps in mL/100 g tissue/min, is chosen for its excellent temporal and spatial resolution for a cerebrovascular target engagement [63]. The expectation is that the effect of psilocybin on regional CBF will supersede the minimal detectable changes in an ASL assessment that is approximately 10 mL/100 g tissue/min for short and longer time spans [64, 65]. ASL-CBF also allows for easier inter-session comparison compared to fMRI [66]. Despite this, there are no studies examining *acute* fMRI or CBF changes immediately following psilocybin administration in MDD. Previous studies conducted by Carhart-Harris et al. [22], as well as Daws et al. [27], stand as sole published works, to our knowledge, examining neuroimaging differences between psilocybin, placebo, and escitalopram in MDD and TRD, respectively, at a *subacute *(i.e., one day after treatment) timepoint.

Blood-based biomarkers can predict diagnosis, response to medications, and elucidate psychedelic therapeutic mechanisms of action. For example, circulating levels of brain-derived neurotrophic factor (BDNF) are decreased in depression and may be altered by psychedelics [38, 39, 67]. Psilocybin’s antidepressant mechanisms may involve BDNF signalling [68], where levels of BDNF are reduced in healthy individuals after receiving psilocybin [69]. Research that examines changes in the presence of BDNF after psilocybin administration may help to confirm its mechanistic role in MDD and in observed neuroplastic changes measured by neuroimaging. C-reactive protein (CRP), an acute-phase reactant that reflects inflammation, is associated with depression severity and response to other rapid-acting antidepressants [70–72]. Elevated CRP levels are associated with depression [70], with capacity to indicate the presence of chronic inflammation [73, 74]. Furthermore, S100 Calcium Binding Protein B (S100B) is increased in MDD and is associated with glial pathology [67]. Assessing changes in S100B post-psilocybin might be important for determining psilocybin’s effect on MDD-related glial pathology. Integrating neuroimaging with blood biomarkers post-psilocybin in MDD remains unexplored and is vital for understanding acute neuroplastic effects and identifying biomarkers of treatment response.

### Objectives {7}

Our primary objective is to test whether psilocybin (prior to adjunctive psychotherapy) leads to significant acute and subacute changes in functional brain activity in networks associated with mood regulation and depression compared to placebo and supportive therapy. We hypothesize that regional CBF will be modulated during the acute phase of treatment relative to placebo, particularly in brain networks associated with mood regulation and depression. Specifically, there will be an acute increase in dorsolateral prefrontal cortex blood flow and a reduction in the subgenual anterior cingulate cortex blood flow between baseline and the first active treatment (Treatment Visit 1). The CBF findings will be contrasted against resting-state fMRI data. Our secondary objective is to examine whether participants treated with psilocybin-assisted psychotherapy will have a greater reduction in depression scores over time signified by the MADRS relative to those who receive placebo. Our secondary hypothesis is that a reduction in MADRS score will be related to an increase in dorsolateral prefrontal cortex blood flow and a decrease in subgenual anterior cingulate cortex blood flow between baseline and the first treatment visit among participants treated with psilocybin compared to placebo. Additionally, our exploratory objective will examine blood biomarkers that are mechanistically related to psilocybin and/or MDD including those involved with increased neurogenesis and neuroplasticity (BDNF), glial pathology (S100B), and systemic inflammation (CRP). It is predicted that these biomarkers will be associated with MADRS scores at baseline. Our exploratory hypothesis is that our baseline blood-based biomarkers of interest will predict treatment response (i.e., changes in depression scores) measured over time by the MADRS. It is expected that there will be a post-treatment increase in BDNF and a reduction in CRP and S100B plasma levels.

### Trial design {8}

**Fig. 1:**
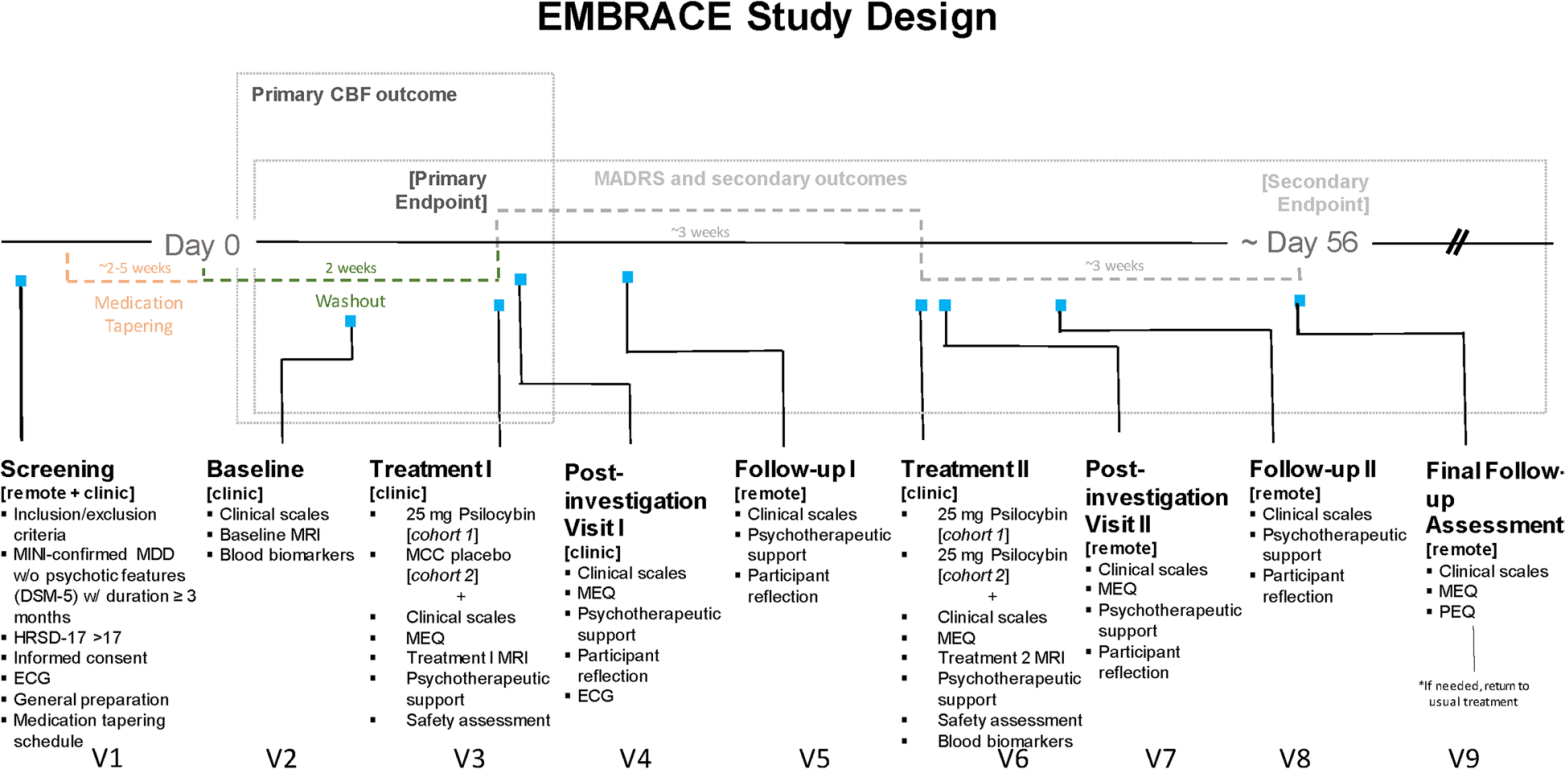
Study design

**Fig. 2:**
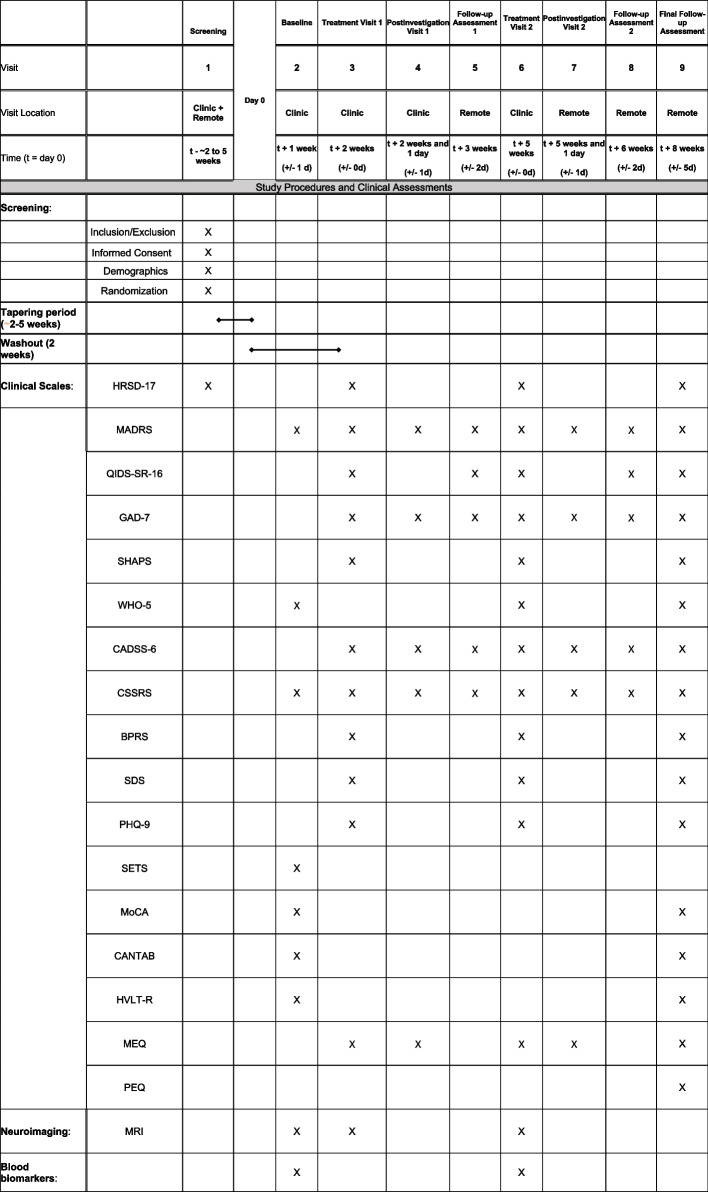
Schedule of events

EMBRACE is a randomized, placebo-controlled, delayed-start trial that investigates neuroimaging, blood biomarkers, and clinical changes associated with psilocybin therapy in those with a unipolar depressive disorder. The protocol is documented as adhering to the SPIRIT checklist [see Additional file 1]. For the purposes of this study, a depressive disorder will include MDD or persistent depressive disorder (PDD) as indicated by the Mini-International Neuropsychiatric Interview (MINI) with a current major depressive episode as defined by the DSM-5.0 and confirmed by an interventional psychiatrist [75]. Study assessments/visits will occur at intervals that are reported in Fig. 2. This study aims to recruit and treat up to 50 participants at Sunnybrook Health Sciences Centre, University of Toronto, Toronto, Ontario, Canada. Participants will be screened by a study coordinator to determine study eligibility, and if eligible will undergo a baseline assessment, preparatory supportive psychotherapy, and neuroimaging 1 week prior to randomization. At the first treatment session (Treatment Visit 1), participants will be randomized to either group 1 or group 2. Participants in group 1 will receive a high dose of synthetic psilocybin (25 mg) while in group 2 will receive inert placebo (25 mg MCC). Three weeks later (Treatment Visit 2), participants in group 1 and group 2 will both receive a high dose of synthetic psilocybin (25 mg). This delayed-start design allows for the comparison of outcomes between the two groups while also evaluating the effect of the intervention over time. At each Treatment Visit, all participants will undergo neuroimaging after dosing, and two qualified staff members will observe and provide supportive psychotherapeutic support for approximately 8 h post-treatment administration. A diagram of the study design and stages is presented in Fig. 1.

## Methods: participants, interventions and outcomes

### Study setting {9}

The study population will be recruited from the Harquail Centre for Neuromodulation, Mood & Anxiety Disorders Programs and general psychiatry clinics at Sunnybrook Health Sciences Centre, University of Toronto, Toronto, Ontario, Canada.

### Eligibility criteria {10}

Fifty participants (> 18 and < 65 years) will be recruited. Potential study participants will have been referred by their circle of care and will undergo pre-screening and confirmation of diagnosis by the study Qualified Investigator (QI), who is an interventional psychiatrist, prior to enrolment in the study. During this assessment, a thorough medical, psychiatric, and medication history assessment will be performed by the QI. Participants will need to have a MINI-confirmed diagnosis of a depressive disorder and meet criteria for a current major depressive episode (MDE) as defined by the DSM-5, which will be confirmed by the QI. Depressive symptoms must be in the moderate-to-severe range at the time of enrolment, as measured by the 17-item Hamilton Rating Scale for Depression (HAMD-17). A 12-lead ECG recording will be obtained to monitor for QTc prolongation and other conditions. Detailed recommendations will be provided for a medication(s) tapering schedule(s) if a participant is taking one or more psychotropic medications prior to study enrolment. Careful monitoring of the participant during a tapering period (~ 2–5 weeks based on tolerability) and during the subsequent 2-week washout period will be done by a study psychiatrist in collaboration with the participant, their primary caregiver, as well as their primary care provider to ensure appropriate down-titration and washout. A washout period will be mandatory if a participant is taking a prescribed antidepressant medication (e.g., SSRIs/SNRIs, tricyclic antidepressants, lithium, MAOIs) and must be undertaken before the trial start date. SSRIs/SNRIs (except fluoxetine), tricyclic antidepressants, antipsychotics, lithium, and monoamine oxidase inhibitors (MAOIs) must be tapered under the direction and supervision of the QI for 2–4 weeks (SSRIs/SNRIs/TCAs), 2–4 weeks (antipsychotics), 4 weeks (Lithium), and 5 weeks (MAOIs and fluoxetine), respectively, and then discontinued prior to receiving the first dose of psilocybin/placebo. The QI will provide detailed instructions to participants for tapering psychotropic medications and will monitor the participant during both the taper and wash out period, prior to the initial dose of psilocybin/placebo [see Additional file 2]. The individualized dosage reduction plan will be developed in collaboration between the QI and the participant, their primary caregiver, and their primary healthcare provider.

Participant enrolment will begin after the screening and tapering period of any medications, but prior to their 2-week washout period. Confirmed enrolment will then trigger randomized allocation of participants to either Treatment Group. Any woman capable of becoming pregnant (e.g., had a menstrual period within the last 12 months) will have their blood analyzed for a βHCG level to rule out pregnancy prior to participating in the study. They will also be required to complete a urine pregnancy test on each treatment day before each treatment administration, and will be counseled to use effective contraception if sexually active during the treatment period.

### Who will take informed consent? {26a}

The study coordinator will have completed all regulatory training in compliance and as mandated by Health Canada and Sunnybrook Research Institute, and the study coordinator will obtain written informed consent from each person that participates in the study. Enrolment will continue until the target sample is reached. Participants will be informed of the background, purpose, procedures, and potential risks or harms associated with the study. It will be expressed to the participant that their participation in the study may be withdrawn at any point during the study without penalty or repercussion.

### Additional consent provisions for collection and use of participant data and biological specimens {26b}

The exploratory aim examines whether blood biomarkers will be associated with treatment responses and thus the informed consent for this study describes the blood draw as an optional data collection. Furthermore, biofluid data collection could be used as part of future studies.

## Interventions

### Explanation for the choice of comparators {6b}

In this randomized, single-center, delayed-start neuroimaging clinical trial, participants will be randomized to receive a high dose of psilocybin (25 mg PO) or MCC (25 mg) in a double-blind manner at the first of two treatment visits. MCC was chosen as the inert placebo as it reduces the potential side effect burden on participants and has no direct effects on cerebrovascular function relative to an active placebo. This choice of placebo will generate positive drug effect expectations during core neuroimaging assessments and has been widely used in many placebo-controlled trials involving psilocybin [22, 27, 76–78].

Three weeks later, all participants, regardless of randomization, will receive psilocybin 25 mg PO. Several phase I and II trials report high dose (25 mg) of psilocybin as having a robust antidepressant effect [23, 79]. The psilocybin dose for this study is reported to be unaffected by age and body mass with respect to total drug exposure and maximum plasma concentration [80] and was comparable to the selective serotonin reuptake inhibitor (SSRI) escitalopram in terms of both efficacy and adverse event frequency [22]. For this study, synthetic psilocybin (25 mg) and inert MCC placebo (25 mg) will be provided by the medical research organization Usona Institute. Both psilocybin and MCC capsules will be individually pre-packaged into high-density polyethylene bottles (25 cc) and labelled with randomized codes in a double-blind manner. MCC is United States Pharmacopeia (USP)-grade encapsulate.

### Intervention description {11a}

There will be a total of two treatment visits on-site at Sunnybrook Health Sciences Centre. For the first treatment visit on day 14, half of the participants (experimental arm) will be administered one 25 mg capsule of psilocybin taken orally with water, while the other half (control arm) will be administered one 25 mg MCC capsule taken orally with water. Three weeks later, all participants will receive one 25 mg capsule of psilocybin taken orally with water during their second treatment visit as a delayed-start measure. Supportive psychotherapy will be provided in conjunction with the consumption of both psilocybin and placebo by two registered psychotherapists licensed by a recognized regulatory body in Ontario (e.g., College of Registered Psychotherapists of Ontario)**.** This therapy will encompass practical support, relaxation techniques, and integration techniques, fostering participants’ openness and curiosity about their experiences. Additionally, unlicensed psychotherapists undergoing licensure may participate in the trial under the direct supervision of a licensed psychotherapist. Following administration of each treatment and their subsequent MRI scan, participants will be guided to a recovery room—a carefully curated therapeutic physical environment that is both esthetically pleasing and conducive to safety—to resume their psychotherapeutic support for the duration of their psychedelic experience until effects have subsided (around 6–8 h post-ingestion). To enhance participants’ positive subjective experiences, the recovery room will have comfortable furniture and soft lighting and resemble a home-like ambiance. Preparatory psychotherapy sessions will be provided beforehand to familiarize participants with the clinical settings, including the recovery room and the route to the MRI scanner. During these sessions, participants will be guided through the procedures that will take place during the study while establishing comfortability, trust, and a positive rapport with the study team.

### Criteria for discontinuing or modifying allocated interventions {11b}

According to the terms of participant safety and voluntary withdrawal, participants who request withdrawal from the study at any time will be immediately withdrawn with no further data collected and the cessation of intervention administration. Participants will not be required to provide reasoning for their withdrawal of consent. However, if reasoning for study withdrawal is voluntarily provided, this will be recorded along with survival data up to the protocol-described follow-up period. Any data collected prior to the withdrawal of consent may be retained and used by Sunnybrook Research Institute. Research staff may also withdraw participants if they are rendered unable to tolerate the trial medication, whether there is a serious adverse event or a persistent adverse event that impedes their daily functioning. The withdrawal procedure also extends to those who are unable or unwilling to follow the study procedures, those who are diagnosed with any medical condition that, in the Qualified Investigators’ opinion, may jeopardize their safety if they were to continue in the trial. If the participant plans to or becomes pregnant during the trial, the QI may stop a participant’s involvement in the study without their consent. If the Sponsor decides to stop the study or if the Regulatory Authority(ies)/research ethics board withdraws permission for the study to continue, the immediate cessation of involvement for all participants will be done without their consent.

### Strategies to improve adherence to interventions {11c}

Participant medication adherence plans will not be necessary as treatment dosing will occur on-site and under the supervision of study personnel responsible for participant care and monitoring. However, participants will be strongly encouraged to speak to the study staff and/or Qualified Investigator should they have any questions or concerns about the study procedures involved.

### Relevant concomitant care permitted or prohibited during the trial {11d}

Concomitant standard-of-care psychiatric interventions and medications that participants would normally receive from their primary care provider/psychiatrist must be discontinued, as described in the study eligibility criteria. To ensure safety and to mitigate any associated risks or adverse effects, participants will follow our antidepressant medication tapering and washout protocol before they begin the study. In alignment with the Human Hallucinogen Research Safety guidelines, any potential adverse events will be mitigated through cultivation of a positive interpersonal atmosphere between the study staff and participants [28]. During the study treatment, and after the MRI scans, supportive psychotherapy will be used to guide and facilitate a calm affect, during a participant’s psychedelic experience, also acting as a de-escalatory method in response to any panic or aggressive behaviour. Although it is highly improbable [16, 21, 81], in the event that a participant experiences moderate agitation and does not respond to the psychological support provided by study staff session, the Qualified Investigator will be notified and authorize the administration of an oral sedative medication, such as sublingual 1 mg lorazepam tablet (considered a rescue medication).

### Provisions for post-trial care {30}

Follow-up assessments will be required after each treatment visit to provide additional psychotherapeutic support and to allow for participant reflection. Participants who have discontinued or completed their participation in the trial will be permitted to resume their usual standard-of-care for their depression symptoms. Participants will be advised in the informed consent that they will need completed paperwork by their general practitioner or psychiatrist to apply to Health Canada’s Special Access Program for compassionate care if they express interest in continuing psilocybin treatment for their depression symptoms beyond the study’s duration. They will also be informed that neither granted access nor affordability can be guaranteed if they pursue this option.

### Outcomes {12}

Exploratory imaging analysis will consist of voxel-wise analyses to fully characterize regional group and session effects. These analyses will probe circuit-based perspectives by considering the following four relevant resting-state networks: (1) salience, (2) left frontoparietal, (3) right frontoparietal, (4) default mode brain regions. Furthermore, we will conduct brain and behavioural responses, namely regional CBF changes in relation to the MADRS scores. Well-validated markers of brain neuroplasticity (BDNF), systemic inflammation (CRP), and glial pathology (S100B) will be measured in blood at baseline and subsequently evaluated as predictors of treatment response.

### Participant timeline {13}

The trial schedule is outlined in Figs. 1 and 2, illustrating the timeline from eligibility screening to final follow-up that includes time points for each treatment visit and follow-up, the clinical scales used, as well as the timing of our primary and secondary outcomes. See participant timeline below for details.

A week into each participant’s washout period, a baseline assessment (day 7) will be conducted, which will include clinical scales, a baseline MRI, and an optional blood biomarker collection, if the participant has indicated their agreement to provide this on the written consent form. One week (day 14) after baseline is the first treatment visit where clinical questionnaires will be completed. Afterwards, participants will be administered either 25 mg of psilocybin or MCC placebo depending on their randomized group allocation. Over the course of 8 h post-psilocybin intake, participants will be monitored every 30 min for routine vitals (HR, BP, %SPO_2_) and dissociative symptoms, using the 6-Item Clinician Administered Dissociative Symptoms Scale and mental status examination by the study coordinator. Approximately 1 h after dosing, participants will undergo a second MRI scan that will include a T1-weighted anatomical scan, ASL and resting-state fMRI sequence with the total scan time lasting approximately 1 h. Participants will then be brought by study personnel to a comfortable room where supportive psychotherapy will be provided. An ECG will be performed within 48 h after treatment administration on the first treatment day. All participants will have a follow-up visit 1 day after the initial treatment that includes relevant clinical scales, psychotherapeutic support, and participant reflection. A second follow-up visit 1 week after the first treatment visit will be remote and will include relevant clinical scales, psychotherapeutic support, and participant reflection. Participants will return to the hospital for open-label treatment with psilocybin 25 mg on the second treatment visit. The QI or delegate will be in-house and available on-call during this time to manage any emergency situations should they arise. Supportive psychotherapy will be provided by a study psychotherapist during each participant’s psilocybin or placebo treatment. Prior to participant discharge, a risk assessment will be performed to ensure that they are safe to leave the center (e.g., risk of harm to self or others and likely serious physical impairment due to their mental state). If they are not deemed safe to leave by study personnel, the QI or delegate will be promptly notified, and the participant will be offered additional time for recovery. If the time exceeds the normal working day, the study QI/delegate will remain available to monitor the participant until the participant’s risk level is reduced and is suitable for discharge home or, if necessary, bring the participant to the emergency department and/or admit directly to the psychiatric unit for further emergency psychiatric care and stabilization.

### Sample size {14}

As this study will employ a longitudinal design with multiple visits where CBF measurements in individual subjects may reasonably be expected to be highly dependent across visits, we will model use linear mixed-effects models of CBF at all visits to account for effects of psilocybin and MCC placebo independently of baseline CBF. Specifically, we will use the following model (in *lme4* syntax):

1 $$CBF\sim Age+Sex+Drug+Drug\;x\;Visit+(\mathit1\;\vert\;ID)$$

To assess the difference in CBF associated with psilocybin relative to placebo, marginal means of a Visit x Drug interaction will be calculated using the *emmeans* package in R, and contrasts between baseline and treatment visit 1 CBF under psilocybin and placebo conditions will be calculated separately, and an additional contrast composed of the difference of these two contrasts will be calculated. Sidak’s correction for multiple comparisons will be used to adjust *p* values. The primary endpoint will be the contrast estimating the between-group difference in within-subject differences in psilocybin- and placebo-induced CBF changes at treatment visit 1 vs baseline (i.e. [V1 psilocybin − V0] − [V1 placebo − V0]).

As a power analysis, we simulate 500 datasets each for groups of size *N* = 5 − 50, in increments of 5. For each simulated subject, baseline CBF is sampled from a normal distribution 55 ± 13 mL/100/g min. At each subsequent visit, a ± 3% test-retest variability is added to the baseline CBF. At psilocybin visits, relative to subject-specific baseline CBF, a proportional 23% ± 23% increase in CBF is added. MCC placebo has no direct psychotropic effects. For each of these 500 datasets, a model (Eq. 1) is fit to the data, and the contrasts described above are calculated. Sufficient power is achieved when evaluation of the between-group contrast of interest for a given group size produce corrected *p*-values below *α* = 0.05 at a frequency equal to or greater than a pre-specified power of 80%. This power analysis suggests two groups of *N* = 20 are sufficient to reject the null hypothesis in > 80% of cases. Sensitivity analyses additionally suggest that sufficient power can be achieved with *N* = 25 in each group if test-retest variability is as high as 7.5%, or if psilocybin effect size and variance is scaled from 23% to as low as 15% [see Additional file 3]. As these simulated datasets are likely somewhat idealized relative to real-world data, we will use a total of *N* = 50 subjects with 25 allocated to each arm.

These anticipated CBF group session differences are consistent with a recently conducted acute neuroimaging clinical trial in bipolar depression [82]. Although we do not conduct a power analysis for H_A,2_, we provide context from the literature in support of the MADRS outcome measure. Example studies incorporating the MADRS outcome measure are provided [see Additional file 4], showing trial design, sample sizes, intervention details, and the reported changes in MADRS score [83–87].

Thus, our MADRS assessments for *N* = 50 is consistent with previous depression trials and support our second hypothesis (H_A.2_).

### Recruitment {15}

As our participant recruitment will solely occur through referral by the multidisciplinary team within the participant’s circle of care at the Harquail Centre for Neuromodulation and the Mood and Anxiety Disorders Clinic at Sunnybrook Health Sciences Centre, particular strategies for participant enrolment will not be required to reach our target sample size.

## Assignment of interventions: allocation

### Sequence generation {16a}

Participants will be randomized with a computer-generated 1:1 randomized allocation into either the psilocybin or placebo group for dosing visit 1 by an independent external assistant using REDCap Randomization Module. Each participant will have an equal probability of being added to either group. Group sizes will be fixed, and study personnel will be blind to randomized allocation. A flowchart for participant randomization is provided [see Additional file 5]. All participants will receive open-label psilocybin at the second treatment visit.

### Concealment mechanism {16b}

The randomized 1:1 allocation sequence will be conducted using the Randomization Module in REDCap software. Each medication bottle label will contain a blinded, unique code that is associated with a specific treatment. Usona Institute will supply a master list containing the correspondence between the blinded, unique codes and their respective treatments. This list will be enclosed within an envelope and securely locked away, inaccessible to all study personnel.

### Implementation {16c}

Participants will be enrolled by the Qualified Investigator upon meeting inclusion criteria (see Table 1) and providing their informed consent. Each bottle of drug product (containing one capsule per bottle) will be labelled by Usona Institute with a tear-away portion denoting the bottle and subject ID to facilitate randomization. The study coordinator will assign a unique identifier to each participant and provide an independent external assistant with subject IDs, bottle IDs, and tear-away portions for randomization. Random assignment to the treatment or control group will be conducted using a computer-generated sequence using REDCap, ensuring equal allocation. The independent external assistant, without involvement from study personnel, will generate the allocation sequence and create a master list of tear-aways concealed in an opaque envelope. Subsequently, the study coordinator will label each bottle according to the randomly assigned subject-bottle ID list.

**Table 1:** Inclusion and exclusion criteria

	**Inclusion criteria**
**Consent**	Must be able and voluntarily willing to provide written informed consent at the screening visit, with reconfirmed verbal consent provided at the beginning of each study visit
**Demographics**	
**Age**	Over 18 and under 65 years old
**Ability**	Able to complete all required assessment tools without assistance or alteration to the copyrighted assessments, as well as to comply with all required study visits
**Responsible Individual/Caregiver**	Must have a responsible individual/caregiver who is able to monitor the participant at home for 24 h after each treatment visit
**Mental health**	
**Psychiatric care**	Must have a psychiatrist and/or general practitioner who is able to provide psychiatric follow-up care
**Diagnosis**	Have a Mini International Neuropsychiatric Interview (MINI)-confirmed diagnosis of a depressive disorder, recurrent or single episode, without psychotic features where the duration of the current episode is at least 3 months Depression of at least moderate severity as defined by a Hamilton Depression Rating Scale (HAMD-17) score of greater than 17
	**Exclusion criteria**
**Physiological health**	
**Medical History**	Uncontrolled or insulin-dependent diabetes Women who are pregnant (self-report or via urine test), nursing, or planning a pregnancy during the timespan of the study History of seizure disorder except for seizures from electroconvulsive therapy and/or febrile seizures in childhood History of stroke, recent myocardial infarction (< 1 year from signing of ICF), uncontrolled hypertension (blood pressure > 140/90 mmHg) or clinically significant arrhythmia within 1 year of signing the ICF Any other clinically significant cardiovascular, pulmonary, gastrointestinal, hepatic, renal or any other major concurrent illness that, in the opinion of the investigator, may interfere with the interpretation of the study results or constitute a health risk for the participant if he/she takes part in the study
**Vital signs**	Abnormal and clinically significant results on a physical examination performed within one month of study participation by a general practitioner, vital signs, ECG, or laboratory test at screening QTc prolongation on ECG defined by > 450 ms in males and > 460 ms in females in V_5_ on a 12-lead ECG [88]
**Lab work**	Positive urine drug screen for illicit drugs or drugs of abuse at screening, a week prior to treatment, and during the trial (any positive urine drug test will be reviewed with participants to determine the pattern of use and eligibility will be determined at the investigator’s discretion) Serial blood counts to achieve a value to meet eligibility — abnormalities in screening/baseline blood work (complete blood counts, electrolyte panel, etc.) will be reviewed by MD, then repeated serially until abnormalities resolve
**Mental health**	
**Current risk**	Any symptoms consistent with psychosis Any symptoms consistent with hypomania and/or mania as assessed by a psychiatrist Other personal circumstances or behaviour judged to be incompatible with establishment of rapport or safe exposure to psilocybin
**Personal history**	Current or past history of bipolar I/II disorder, schizophrenia, schizoaffective disorder, psychotic disorder, or delusional disorder as assessed by a structured clinical interview (MINI) ≥ 1 suicide attempt in the past year requiring hospitalization, defined using the Columbia Suicide Severity Rating Scale (CSSRS) (Q6 (past year) = “y”) and clinical interview with a psychiatrist History of substance use and/or alcohol use disorder, of moderate severity or greater, in the past 12 months Lifetime history of substance use disorder with a hallucinogen Lifetime history of substance-induced psychosis Depression secondary to other medical conditions or bipolar I and II disorder
**Family history**	Family history of a first degree relative with a diagnosis of schizophrenia or a primary psychotic disorder and/or bipolar disorder
**Substance use**	Exposure to psilocybin or any other psychedelic in the past 12 months prior to screening and/or during the current MDE and use of psychedelics, such as ayahuasca/LSD, during the current depressive episode
**Diagnosis**	A clinical diagnosis of antisocial personality disorder and/or paranoid personality disorder (defined as meeting DSM-5.0 criteria) based on clinical interview and the MINI 7.0. Positive diagnoses on the MINI will be subject to confirmation at a clinical interview by a psychiatrist An active clinical diagnosis of borderline personality disorder as confirmed by the MINI 7.0 Diagnosis of any mild or major neurocognitive disorder meeting DSM-5 criteria and based on clinical interview/cognitive screening by a psychiatrist
**Other**	Current enrolment in an interventional study for depression or participation in such within 30 days of screening

## Assignment of interventions: Blinding

### Who will be blinded {17a}

This is a double-blinded study where neither the participant, their usual health care providers, nor the research team (including the QI/PI) will be explicitly aware of participants’ group assignment. Randomization is performed by a computer-generated sequence and both the psilocybin and placebo will be encapsulated and labelled in a double-blind manner with associated randomized codes.

### Procedure for unblinding if needed {17b}

In exceptional cases, the blind may be broken if the QI has deemed it medically necessary or if a data freeze has occurred based on the mutual discretion and agreement between PI and QI. In this instance, the QI and one SHSC staff representative (who is not involved in the trial in any capacity), will have access to the master key containing the blinded, unique bottle codes associated with a treatment and the unblinded participants’ data. Details pertaining to the unblinding that include the date and reason for unblinding will be documented in the CRF.

## Data collection and management

### Plans for assessment and collection of outcomes {18a}

The assessments to be collected for both primary and secondary outcomes can be found in Table 2. For each participant, much of the data will be securely stored in the form of an electronic case report form (eCRF) which will include participant demographics, clinical scales and assessments, medical information including any pre-existing conditions and history, concomitant medications and therapies, vital signs (i.e., ECG, blood pressure), baseline blood tests, follow-up visits, any AEs or SAEs (if applicable), documentation of a participant’s informed consent, study visit dates, participant identification numbers, and any documentation pertaining to the appropriate data entry, management, validation, and archival. Paper-based copies of study questionnaires will be kept in the physical CRF with the unique, anonymized identification number assigned to a given participant and securely stored on-site in an area with restricted access at the hospital. Participant data captured during the trial will only be identified by a participant ID and managed in the electronic CRF (eCRF) that will be securely stored in online Research Electronic Data Capture (REDCap) software. REDCap is a secure, web-based software that is designed to support data capture for clinical trials and research studies. MRI data will be stored on a secure research server at Sunnybrook Health Sciences Centre. All questionnaires used in the study have been previously published and validated for research and/or clinical use. The ASL and MRI techniques have been previously published and validated.

**Table 2:** Primary and secondary outcomes

**Primary outcomes**	• Significant alterations in regional CBF across three a priori brain regions from baseline to treatment visit 1
	• Improvement in depressive symptoms using the Montgomery-Asberg Depression Rating Scale (MADRS) from baseline to week 3 and week 6 following initial psilocybin/placebo administration. Higher scores with respect to the MADRS minimum (0) and maximum (60) values represent a worse treatment outcome [89]
**Secondary outcomes**	• Baseline grid-version of the 17-item Hamilton Depression Rating Scale (GRID-HAMD-17) score [Time Frame: Baseline]. The GRID-HAMD is a 17-item clinician-administered rating scale designed to assess severity of depressive symptoms. The score range is 0 to 52, with higher score indicating more severe depression [90]
	• Incidence of response [Time Frame: Up to study end point]. The proportion of participants with a response (defined as a ≥ 50% improvement in MADRS total score from Baseline) at week 3 post psilocybin administration. The minimum and maximum values are 0 and 60 and a higher score represents a worse outcome [91]
	• Incidence of remission [Time Frame: study endpoint]. The proportion of participants with remission (defined as MADRS total score < 11) at week 3 and 6 following the initial psilocybin/placebo administration. The minimum and maximum values are 0 and 60 and a higher score means a worse outcome [91, 92]
	• Patient Health Questionnaire 9-item (PHQ-9) [Time Frame: Baseline up to study end point/weeks]. The PHQ-9 is a self-rated measure of depressive symptom severity in the past two weeks. Each of the nine items is rated on a Likert scale, ranging from 0 (not at all) to 3 (nearly every day), and summed for a total score between 0 (no symptoms) to 27 (most severe) [93]
	• 16-item Quick Inventory of Depressive Symptomatology–Self-Report (QIDS-SR-16) [Time Frame: Baseline up to study end point/weeks]. The QIDS-SR-16 is a self-report scale with scores that range from 0 to 27. Higher scores indicate greater depression [94]
	• Clinical Global Impressions Scale (CGI) [Time Frame: Baseline up to study end point/weeks]. The CGI severity module assesses the severity of a person’s depressive illness using a seven-point Likert scale, ranging from “Normal, not at all depressed” to “Among the most extremely depressed patients”. The CGI improvement module evaluates the global improvement of a person’s condition since their last visit on a seven-point Likert scale, ranging from “Very much improved” to “Very much worse” [95]
	• Columbia Suicide Severity Rating Scale (CSSRS) [Time Frame: Baseline up to study end point /weeks] The CSSRS evaluates suicidal ideation and behaviour. The suicidal ideation score ranges from 0 (no ideation) to 5 (active suicidal ideation with specific plan and intent). Suicidal ideation intensity score ranges from 0 (no ideation) to 25 (most severe). The presence of suicidal behaviour is rated as a binary response; the lethality of previous actual attempts is rated on a scale of 0 (no or very minor physical damage) to 5 (death) and the potential lethality of actual attempts are rated on a scale of 0 (behaviour not likely to result in injury) to 2 (behaviour likely to result in death despite available medical care) [96]
	• Brief Psychiatric Rating Scale (BPRS) [Time Frame: Baseline up to study end point /weeks]. The BPRS rating scale has 18 items, each item rated on a severity scale of 1 (not present) to 7 (extremely severe). 0 is entered if the item is not assessed [97]
	• Sheehan Disability Scale (SDS) [Time Frame: Baseline up to study end point /weeks]. The SDS total score ranges from 0 to 30 with 0 representing no impairment and 30 representing severe impairment. The last two items of the scale (Days Lost and Days Unproductive) range from 0 to 7 (higher number denotes greater impairment) [98]
	• World Health Organization-5 Well-Being Index (WHO-5) [Time Frame: Baseline up to study end point/weeks]. The WHO-5 is a measure of overall well-being, rated on a scale of 0 to 25, with higher scores denoting higher quality of life [99]
	• World Health Organization Disability Assessment Schedule 2.0 (WHODAS 2.0) [Time Frame: Baseline up to study end point/weeks]. The WHODAS 2.0 is a self-reported disability questionnaire based on the International Classification of Functioning, Disability, and Health (ICF). It includes 36 questions, organized under six domains (cognition, mobility, self-care, getting along, life activities and participation). Each question must be answered based on the perceived difficulty for performing activities using a 5-point scale (none, mild, moderate, severe, and extreme) [100]
	• Generalized Anxiety Disorder-7 (GAD-7) [Time Frame: Baseline up to study end point/weeks]. Total score ranges from 0 to 21; a higher score denotes greater symptom severity [101]
	• Snaith-Hamilton Pleasure Scale (SHAPS) [Time Frame: Baseline up to study end point/weeks]. The SHAPS total score ranges from 14 to 56, wherein a higher score indicates greater hedonic capacity (lower anhedonic severity) [102]
	• 6-Item Clinician Administered Dissociative Symptom Scale (CADSS-6) [Time Frame: Treatment visit 1 up to study end point/weeks]. Total scores range from 0 to 16, wherein a higher score indicates greater dissociation [103]
	• Stanford Expectations of Treatment Scale (SETS) [Time Frame: Baseline] This scale contains six items measuring positive (3 items) and negative (3 items) treatment expectancies. Each of the six items is coded with a similar 7-point scale starting from “strongly disagree” to “strongly agree” [104]
	• Montreal Cognitive Assessment (MoCA) total score [Time Frame: Baseline up to study end point]. Cognitive Screening assessment tool [105]
	• We will use the CANTAB software program for evaluating cognitive domains. [Time Frame: Baseline up to study endpoint]. Domains to be assessed: Sustained attention (Rapid Visual Information Processing), Psychomotor speed (Reaction Time), Executive function (Spatial Working Memory, One Touch Stockings of Cambridge), Memory (Delayed Matching to Sample, Paired-associates learning), and Emotional cognition (Emotion Recognition Task) [106]
	• Hopkins Verbal Learning Test-Revised (HVLT-R) Total Score [Time Frame: Baseline up to study endpoint]. A list learning test that contains 12 nouns that are read to a participant for three consecutive trials. After each trial, a participant is asked to recall the words that were read to them. The number of words recalled on each trial is summed together to produce a total score. The higher total score equates to a better outcome [107]
	• Revised Mystical Experience Questionnaire (MEQ) [Time Frame: 6–8 h post-treatment, the day after treatment, and at Final follow-up]. A validated self-report revised 30-item questionnaire recording elements that comprise the mystical experience [108]
	• Persistent Effects Questionnaire (PEQ) [Time Frame: Final follow-up]. A non-validated self-report 145-item questionnaire that describe any changes in the participants’ lives that may be attributed to the psilocybin treatment [108]

### Plans to promote participant retention and complete follow-up {18b}

Participants will be compensated for their commitment to all evaluations and training sessions in the form of $75 CAD per session until their final follow-up that indicates their last session.

### Data management {19}

Any data collected in this study by appropriate study personnel, including those involved in data entry, will be recorded as source documents, and further entered into both paper and electronic case report forms in an anonymized manner. Any essential documents will be collected and stored in the Trial Master File for this study in accordance with Division 5 Regulations and ICH GCP E6. Storage of data will be centrally stored and managed by the Centre for Clinical Trial Support at SHSC. Contemporaneous review of the laboratory results and the assessment of clinical significance for those results considered out of range will be documented by means of dated signature by the reviewing investigator. Those who are given authorization to access the data will be determined by the study investigators. The investigators will review individual study participant records and append a signature where appropriate to ensure that data is accurate, complete, and secure.

### Confidentiality {27}

Confidentiality will be maintained for all potential and enrolled participants in accordance with the requirements stipulated by the Canadian Personal Health Information Protection Act of 2004 (PHIPA) and the Sunnybrook Health Sciences Centre Research Ethics Board (REB). Any personal information collected and stored in paper-based files will be kept in a secure area within SHSC; however, many of the eCRFs will be stored in a secure REDCap web-based application.

### Plans for collection, laboratory evaluation, and storage of biological specimens for genetic or molecular analysis in this trial/future use {33}

Baseline blood-based biomarkers that are mechanistically related to psilocybin and/or MDD will be collected, made optional in the consent form, and will be investigated for potential association with MADRS scores at baseline. Peripheral biomarker levels will be evaluated for associations with treatment response (changes in MADRS scores) over time. Blood plasma and serum samples will be stored and batched on-site and in the appropriate − 80 °C freezer at SHSC for future analysis.

## Statistical methods

### Statistical methods for primary and secondary outcomes {20a}

The primary and secondary analyses will be conducted at Sunnybrook Health Sciences Centre, using open-source software tools, to the extent it is possible. Image processing will be conducted by a trained individual who will remain blind to the treatment allocation throughout the data analysis period. We propose to analyze our primary outcome using mixed-effects linear regression to measure CBF changes across our regions of interest from Baseline to Treatment Visit 1, as well as MADRS changes from Baseline, Week 3, and Week 6 following administration. A priori CBF regions consist of emotion regulation regions that are disrupted in MDD [109, 110], comprising the dorsolateral prefrontal cortex, salience network regions comprising the insula cortex, and default mode network comprising the parietal precuneus.

We will determine an optimal model for data analysis by fitting the model described in Eq. 1 to data using the *lme4* package in R, as well as additional models adding interaction effects of age, sex, and number of prior failed antidepressant treatments with drug to assess whether demographics or treatment-resistance modify the effect of psilocybin. An optimal model will be selected by comparing models fit by maximum likelihood estimation using a chi-squared test of model deviance. This will provide the added benefit of further robustness against the possibility of model non-convergence. As described in the sample size calculation, marginal means of a Visit x Drug interaction will be estimated using the *emmeans* package in R, and two within-subject contrasts of baseline to treatment visit 1 change in CBF will be calculated as well as a between-subject difference in these contrasts. Sidak’s correction for multiple comparisons will be applied to adjust *p*-values, with the primary outcome measure being the between-subject difference in the change in CBF attributable to psilocybin vs placebo at treatment visit 1.

For our exploratory analysis, peripheral levels of S100B, BDNF, and CRP will be measured using standard enzyme-linked immunosorbent assays or similar multiplexed immunoassay. All data analyses will be performed using SPSS 22.0. Multivariate linear regression analysis will be used to assess the relationship between baseline and change in peripheral biomarker measures and post-treatment change in MADRS scores.

### Interim analyses {21b}

N/A. We do not have a planned interim analysis for this study.

### Methods for additional analyses (e.g., subgroup analyses) {20b}

We will perform sensitivity analyses to evaluate the relationship between sex and the primary outcome measures. No additional analyses are planned.

### Methods in analysis to handle protocol non-adherence and any statistical methods to handle missing data {20c}

In the case(s) where a participant drops out of the study after receiving their first dose of placebo/psilocybin then the last observation/data will be included in an intention-to-treat analysis.

### Plans to give access to the full protocol, participant-level data, and statistical code {31c}

Once the primary and secondary a priori endpoint data has been reported, we intend to share our data upon written request with qualified investigators. Data sharing agreements will be in place.

## Oversight and monitoring

### Composition of the coordinating center and trial steering committee {5d}

As this is an investigator-initiated, single-center study, the Trial Steering Committee will be composed of the study’s principal investigators, Dr. Sean M. Nestor (SMN) and Dr. Bradley J. MacIntosh (BJM) who will meet once a month to review the study’s progression, its safety data, and ensure adherence to ethical and regulatory standards. The study coordinator will act as the coordinating center for this study, ensuring daily management of the trial that includes appropriate data management, maintaining the blind, safety reporting, statistical analysis of data, communication between the study team and regulatory authorities, and regulatory compliance. The daily activities required for the study will be carried out by the QI, study coordinator, study psychotherapist, and research assistants, who will also meet weekly to discuss study progression.

### Composition of the data monitoring committee, its role and reporting structure {21a}

For this trial, two psychiatrists at Sunnybrook Health Sciences Centre who are not directly involved with the study, with no financial relationship with the study, will serve as independent medical monitors (IMMs) for the study. The IMMs will have relevant expertise with psychopharmacology and will have the primary responsibility to provide independent safety monitoring in a timely fashion. This will be accomplished by reviewing adverse events, immediately after they occur or are reported, with follow-up through resolution. During the trial, the monitors will evaluate individual and cumulative participant data and provide recommendations regarding the safe continuation of the study.

### Adverse event reporting and harms {22}

Reporting of adverse events, including adverse drug reactions, will be recorded in the case report form/electronic case report form to be reviewed by the QI. If any AEs are deemed serious adverse events (SAEs) or serious unexpected adverse drug reactions (SUADRs), they will be recorded and promptly reported to the Sunnybrook REB and Health Canada in accordance with Division 5 of the *Food and Drug Regulations* and local reporting requirements and timelines.

### Frequency and plans for auditing trial conduct {23}

All study-related source data/documents will be made directly available to the IMMs and regulatory authorities including Health Canada and Sunnybrook REB when a formal audit, regulatory inspection, and/or trial-related monitoring is performed. All data that is collected will be accurate and verifiable by source documents using a clinical data management standard operating procedure. Quality assurance and control systems will be in accordance with ICH-GCP and local regulations.

### Plans for communicating important protocol amendments to relevant parties (e.g., trial participants, ethical committees) {25}

For any protocol amendments, study investigators will be notified of any changes made after mutual agreement of the proposed changes have been established across investigators. The Sunnybrook REB will be notified through the submission of a request form for protocol amendments. For any amendments that may affect participants involved in the study, these changes will be communicated to each participant once they have been made, to which we will look to obtain an updated informed consent. A completely new REB submission will be made for any substantial changes to the protocol.

### Dissemination plans {31a}

The results and experiences of this trial will be jointly published by the investigators in peer-reviewed medical and scientific journals. Ad hoc writing and publication committees will be defined for writing and reviewing publications. The support of the sponsor will be recognized in all publications that result from this study.

## Discussion

The EMBRACE trial will be the first to examine the real-time and subacute neuroplastic effects of high-dose synthetic psilocybin (25 mg) versus placebo in MDD using CBF and fMRI. Employing a non-psychoactive placebo and a delayed-start design, the study conducts serial MR with ASL perfusion-MRI and BOLD-fMRI sequences at baseline and 1-h post-dosing for each treatment visit. It aims to compare acute (intraday) CBF changes, psilocybin verses placebo response, subacute network changes, and cumulative treatment effects. While landmark clinical studies have demonstrated promising response rates with assisted psychotherapy, important drug mechanistic studies are lagging the enduring enthusiasm and emerging usage among the public [111].

Our protocol innovates several key aspects. Firstly, to our knowledge, only a few studies have integrated the combination of ASL perfusion and resting-state fMRI into their study design to examine CBF differences within healthy [33, 112] and treatment-resistant depression [113] populations. To address these gaps, this trial uses BOLD-ASL fMRI at three time points to acutely and sub-acutely capture the robust CBF hemodynamic responses to psilocybin in comparison to placebo in MDD. Secondly, a relatively high dose (25 mg) of psilocybin was chosen based on prior efficacy in depression and safety profiles [21, 114, 115]. Thirdly, the trial includes a tailored tapering period for conventional antidepressants to maximize participant safety and accurately characterize psilocybin-evoked brain activity. Additionally, this trial incorporates a comprehensive battery of clinical measures, including serial MADRS assessments, pre- and post-treatment, as well as follow-up time points to establish treatment efficacy, durability of clinical response, and exploratory relationships with neuroimaging measures. The brain-behaviour relationship as exhibited by both CBF alterations and MADRS scores will also be examined in this trial, including our blood biomarkers of interest that will complement the BOLD/ASL fMRI data.

Upon completion of the proposed study, our findings aim to establish psilocybin’s efficacy as a novel antidepressant therapy while elucidating its effect on brain networks associated with depression. Our hope is that the proposed study aims to inform decision-making and future treatment protocols in addressing the high prevalence of MDD by understanding the relevant mechanisms by which psilocybin exerts its therapeutic effects, ultimately contributing to more efficient treatment delivery and scalability.

## Limitations of the trial design

Anticipated limitations of our trial design include its single-center design and modest sample size, potentially limiting generalizability. To enhance statistical power, we have incorporated three imaging time points to improve our ability to detect intra-subject changes and focused our primary neuroimaging analysis on a priori brain regions linked to MDD and psilocybin’s effects.

## Trial status

The protocol version 4.7 was approved on 10 October 2023 by the Sunnybrook Health Sciences Centre REB. Recruitment is expected to commence in August 2024, and is expected to be completed by August 2027.

### Supplementary Information

Supplementary Material 1.

Supplementary Material 2.

Supplementary Material 3.

Supplementary Material 4.

Supplementary Material 5.

## Data Availability

Once data entry is complete and publication is finalized, access to the final trial dataset with deidentified participant data will only be available to the study investigators and coordinator. Deidentified safety data will be provided to Usona Institute in accordance with a formal agreement.

## Abbreviations

5-HT: 5-Hydroxytryptamine
AE: Adverse event
ASL: Arterial spin labelling
BDNF: Brain-derived neurotrophic factor
BOLD: Blood oxygenation level-dependent
CBF: Cerebral blood flow
CRF/eCRF: Case report form/electronic case report form
CRP: C-Reactive Protein
CSSRS: Columbia Suicide Severity Rating Scale
DMN: Default Mode Network
DSM-5.0: Diagnostic and Statistical Manual of Mental Disorders 5.0
ECG: Electrocardiogram
fMRI: Functional magnetic resonance imaging
GCP: Good Clinical Practice
HAMD: Hamilton Rating Scale for Depression
ICF: Informed consent form
ICH: International Council for Harmonisation
IMM: Independent Medical Monitor
LSD: Lysergic acid diethylamide
MADRS: Montgomery-Åsberg Depression Rating Scale
MAOI: Monoamine oxidase inhibitor
MDD: Major depressive disorder
MDE: Major depressive episode
MINI: Mini International Neuropsychiatric Interview V-5.0.0
mPFC: Medial prefrontal cortex
MR/MRI: Magnetic resonance/magnetic resonance imaging
PCC: Posterior cingulate cortex
PDD: Persistent depressive disorder
PHIPA: Personal Health Information Protection Act
PI: Principal Investigator
PO: Per os/by mouth
QI: Qualified Investigator
QTc: Q-T corrected
REB: Research Ethics Board
S100B: S100 Calcium Binding Protein B
SAE: Serious adverse event
SHSC: Sunnybrook Health Sciences Centre
SNRI: Serotonin and norepinephrine reuptake inhibitor
SPO2: Oxygen saturation
SPSS: Statistical Package for the Social Sciences
SPIRIT: Standard Protocol Items—Recommendations for Interventional Trials
SSRI: Selective serotonin reuptake inhibitor
SUADR: Serious unexpected adverse drug reaction
TCA: Tricyclic antidepressants
USP: United States Pharmacopeia
