# Supplementary material for: Engaging Mood Brain Circuits with Psilocybin (EMBRACE): a study protocol for a randomized, placebo-controlled and delayed-start, neuroimaging trial in depression
Source: Trials. 2024 Jul 3;25:441. doi: 10.1186/s13063-024-08268-6 (PMC11221029; doi:10.1186/s13063-024-08268-6)
Supplement: Supplementary file 2 — Supplementary Material 2. [file 13063_2024_8268_MOESM2_ESM.pdf]

Date ____________ ID _________

EMBRACE MEDICATION TAPERING INSTRUCTIONS

1. Basic information

- This instruction form, a part of the eligibility screening, outlines the tapering schedule for any psychotropic medication use prior to your participation in the study.

  Please review this form carefully as it outlines the tapering timelines depending on the kind of prescribed psychotropic medication that you currently use.

2. Screening

- For your participation in this study, along with this tapering schedule, you must:
  - Meet inclusion criteria,
  - Have a confirmed Major Depressive Disorder with moderate-to-severe Major Depressive Episode,
  - Provide informed consent,
  - Complete an electrocardiogram (ECG).

3. Medication-dependent Tapering Schedule

Your **dosage reduction plan** will be made with you by the study doctor (Dr. Sean Nestor) in conjunction with your primary healthcare provider.

	Medication type	Timeline
☐	Selective serotonin reuptake inhibitors (**SSRIs**) (e.g., *Citalopram*, *Fluoxetine*, *Escitalopram*, *Sertraline*, etc.),<br><br>Serotonin and norepinephrine reuptake inhibitors (**SNRIs**) (e.g., *Desvenlafaxine*, *Duloxetine*, *Venlafaxine*, *Levomilnacipran*, etc.),<br><br>&<br><br>Tricyclic antidepressants (**TCAs**) (e.g., *Amitriptyline*, *Amoxapine*, *Desipramine*, *Doxepin*, etc.)	**2 to 4 weeks**
☐	**Antipsychotics** (e.g., *Risperidone*, *Quetiapine*, *Olanzapine*, *Ziprasidone*, etc.)	**2 to 4 weeks**
☐	**Lithium**	**4 weeks**
☐	Monoamine Oxidase Inhibitors (**MAOIs**) (e.g., *Isocarboxazid*, *Phenelzine*, *Selegiline*, *Tranylcypromine*, etc.)	**5 weeks**

Date ____________ ID ________

A **washout period** of **2 weeks** must occur immediately after your timeline noted above. This period is when no medications are taken.

*All female participants must take a pregnancy test before enrolment in this study.

4. Support and Emergency

- Close monitoring of your tapering timeline and washout period will be done by Dr. Nestor along with your primary caregiver and primary healthcare provider.

  Your safety and well-being are our utmost priority: If you have any concerns or if you are experiencing any adverse effects from the reductions in medication, **please notify your primary healthcare provider, primary caregiver, and the study doctor as soon as possible**. Dr. Nestor's contact information is found below along with additional support resources that are available:

  Sean Nestor, MD, PhD, FRCPC

  Office phone: (416) 480-6100 ext. 4085

  Email: sean.nestor@sunnybrook.ca

  ****If you are IN CRISIS or it is a serious emergency requiring immediate attention, please:**

  1. **Contact emergency services by dialing 911,**

     and/or,

  2. **Travel to your nearest hospital emergency department.**

Additional resources:

Suicide Crisis Helpline: Call or Text 9-8-8

Help is available 24 hours a day, 7 days a week. This helpline offers support that is bilingual, trauma-informed, culturally appropriate, and available to anyone in Canada.

Talk Suicide: 1-833-456-4566 or talksuicide.ca
Offers toll-free support to people in Canada who have concerns about suicide. Phone line available 24/7 or text 45645 between 4 p.m. and midnight ET.

ConnexOntario: **1-866-531-2600 or connexontario.ca**

Free and confidential health services information for people experiencing problems with alcohol and drugs, mental illness or gambling. This website also has a live online chat.
