# Supplementary figures and images for: Engaging Mood Brain Circuits with Psilocybin (EMBRACE): a study protocol for a randomized, placebo-controlled and delayed-start, neuroimaging trial in depression
Source: Trials. 2024 Jul 3;25:441. doi: 10.1186/s13063-024-08268-6 (PMC11221029; doi:10.1186/s13063-024-08268-6)

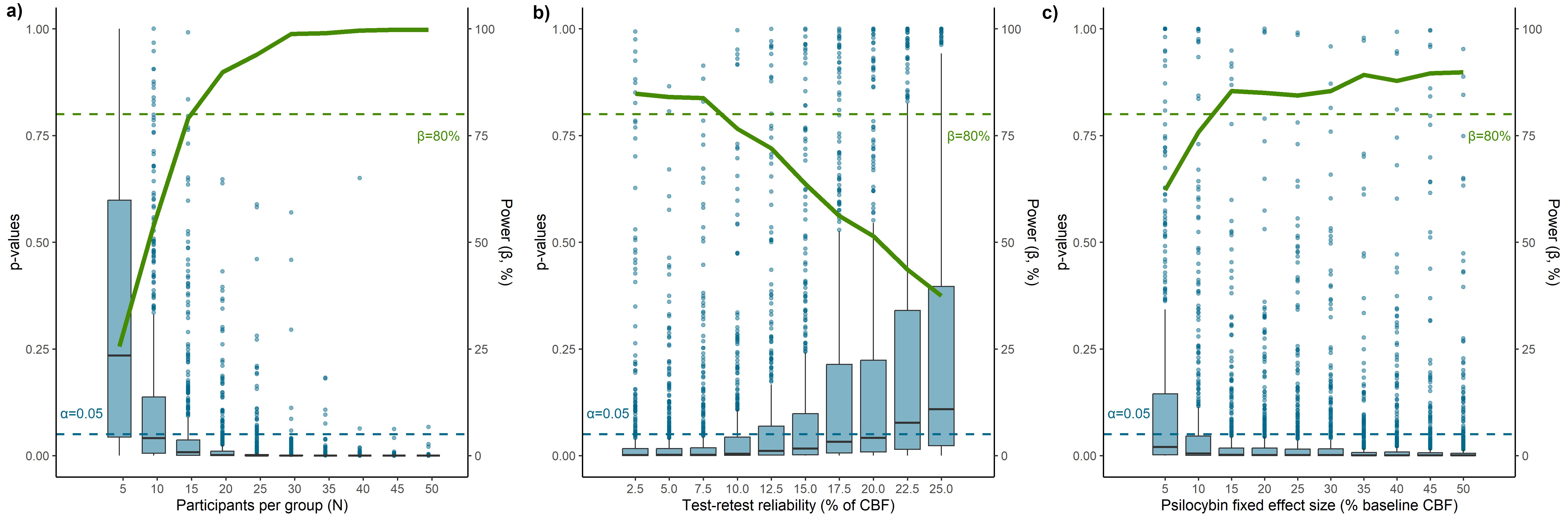

Supplement: Supplementary file 3 — Supplementary Material 3. [file 13063_2024_8268_MOESM3_ESM.png]

## CONSORT Participant Randomization Flowchart

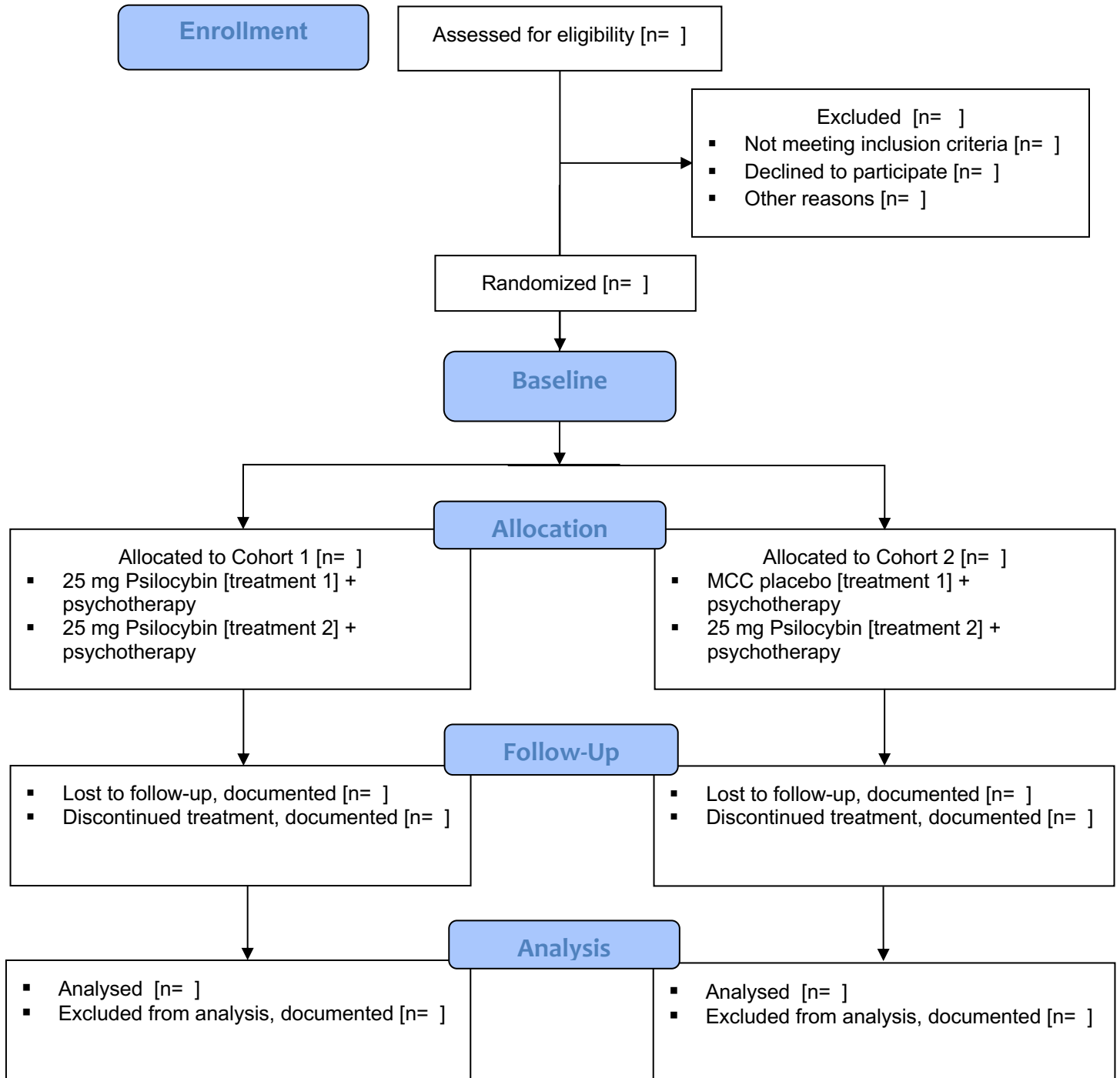

Supplement: Supplementary file 5 — Supplementary Material 5. [file 13063_2024_8268_MOESM5_ESM.pdf]
