# Supplementary material for: Engaging Mood Brain Circuits with Psilocybin (EMBRACE): a study protocol for a randomized, placebo-controlled and delayed-start, neuroimaging trial in depression
Source: Trials. 2024 Jul 3;25:441. doi: 10.1186/s13063-024-08268-6 (PMC11221029; doi:10.1186/s13063-024-08268-6)
Supplement: Supplementary file 4 — Supplementary Material 4. [file 13063_2024_8268_MOESM4_ESM.pdf]

PMID	Design	Participants	Interventions	∆MADRS at 24 h	Other
24821196	Randomized, double-blind, cross-over	18 adults with MDD	50 mg of ketamine hydrochloride (saline placebo comparator)	Mean difference between ketamine and placebo was 7.6 ± 3.7 (95% CI = 3.9 to 11.3)	Response rates at 24 h of 8/18 following ketamine and 1/18 following placebo (p = 0.033)
29656663	Randomized, double-blind, parallel arm	66 adults with MDD and suicidality	84 mg of esketamine twice weekly for 4 weeks (placebo comparator)	Least-squares mean difference between esketamine and placebo was 7.5 (SE = 3.29, p = 0.029)	Remission rates at 24 h of 12/35 following esketamine and 5/31 following placebo
23803871	Randomized, double-blind, cross-over	27 adults with unipolar depression	0.54 mg/kg for 30 min (placebo comparator)	Mean difference between ketamine and placebo was 5.7 (95% CI = 1.8 to 6.2)	Response rate at 24 h of 10/27 following ketamine and 1/27 following placebo
23982301	Randomized, double-blind, parallel arm	73 adults with TRD	0.5 mg/kg of ketamine hydrochloride for 40 min (0.045 mg/kg midazolam comparator)	Mean difference between ketamine and midazolam was 7.95 (95% CI = 3.20 to 12.71)	Response rate at 24 h of 64% following ketamine and 28% following midazolam
29903051	Randomized, double-blind, parallel arm	29 adults with TRD	1 mL/kg of ayahuasca adjusted to contain 0.36 mg/kg of N-dimethyltryptamine (placebo comparator)	Mean difference between ayahuasca and placebo was 14.9	Response rate at 24 h of 50% following ayahuasca and 46% following placebo
